# Daptomycin in experimental murine pneumococcal meningitis

**DOI:** 10.1186/1471-2334-9-50

**Published:** 2009-04-30

**Authors:** Barry B Mook-Kanamori, Mark S Rouse, Cheol-In Kang, Diederik van de Beek, James M Steckelberg, Robin Patel

**Affiliations:** 1 Division of Infectious Disease, Department of Internal Medicine, College of Medicine, Mayo Clinic, Rochester, MN, USA; 2 Division of Clinical Microbiology, Department of Laboratory Medicine and Pathology, College of Medicine, Mayo Clinic, Rochester, MN, USA

## Abstract

**Background:**

Daptomycin, a lipopeptide antibiotic, could be an alternative to vancomycin for treatment of pneumococcal meningitis. We determined the activity of daptomycin versus vancomycin, with dexamethasone as an adjuvant, in a murine model of pneumococcal meningitis.

**Methods:**

Ninety-six 25–30 gram mice were inoculated intracisternally with serotype 3 *Streptococcus pneumoniae *modified by the integration of a luminescent *lux *operon. All mice were treated with either dexamethasone 1 mg/kg intraperitoneally every 6 hours alone or in combination with either vancomycin or daptomycin, also administered intraperitoneally. Serum antimicrobial concentrations were selected to approximate those achieved in humans. Following treatment, bioluminescence and cerebrospinal fluid (CSF) bacterial concentrations were determined. Caspase-3 staining was used to assess apoptosis on brain histopathology.

**Results:**

Sixteen hours post intracisternal inoculation, bacterial titers in CSF were 6.8 log_10 _cfu/ml. Amongst the animals given no antibiotic, vancomycin 50 mg/kg at 16 and 20 hours or daptomycin 25 mg/kg at 16 hours, CSF titers were 7.6, 3.4, and 3.9 log_10 _cfu/ml, respectively, at 24 hours post infection (p-value, < 0.001 for both vancomycin or daptomycin versus no antibiotic); there was no significant difference in bactericidal activity between the vancomycin and daptomycin groups (p-value, 0.18). CSF bioluminescence correlated with bacterial titer (Pearson regression coefficient, 0.75). The amount of apoptosis of brain parenchymal cells was equivalent among treatment groups.

**Conclusion:**

Daptomycin or vancomycin, when given in combination with dexamethasone, is active in the treatment of experimental pneumococcal meningitis.

## Background

Pneumococcal meningitis is associated with high mortality and morbidity rates [1]. Patients with suspected or proven pneumococcal meningitis should receive immediate empirical therapy consisting of a combination of a third-generation cephalosporin and vancomycin, plus dexamethasone [1].

Daptomycin, a lipopeptide antibiotic which has excellent activity against a broad range of Gram-positive microorganisms, including penicillin and cephalosporin resistant pneumococci [2,3], could be an alternative to vancomycin for treatment of pneumococcal meningitis. Rapid bactericidal activity, without lysis and limited inflammatory response, makes daptomycin an attractive possibility for the treatment of multidrug-resistant pneumococcal meningitis [2,3]. No published animal studies have evaluated the combination of daptomycin plus dexamethasone for treatment of experimental pneumococcal meningitis.

In this study, we used a murine model of pneumococcal meningitis to compare the activity of daptomycin with that of vancomycin, when given in combination with dexamethasone.

## Methods

### Bacteria

*Streptococcus pneumoniae *A 66.1 serotype 3 rendered bioluminescent by integration of a modified *lux *operon into its chromosome (*S. pneumoniae *Xen 10, Xenogen Corporation, Alameda, CA) was studied. This strain has been previously shown to be as virulent as its parent strain [4]. Serotype 3 is one of the most common pneumococcal serotypes causing community-acquired bacterial meningitis in adults [5]. Bacteria were incubated in Todd-Hewitt broth (THB) at 37°C in 5% CO_2 _to an OD_620 _of 0.4 and subsequently rinsed in phosphate buffered saline (PBS) and centrifuged to yield a final bacterial concentration of 5 × 10^10 ^colony forming units (cfu)/ml. The exact number of cfu in the inoculum was determined retrospectively by growth of serial dilutions of the inoculum on blood agar plates.

### In vitro studies

The MBC and MIC of daptomycin and vancomycin (with and without dexamethasone), and of penicillin, against *S. pneumoniae *Xen10 were determined using methods described by the Clinical Laboratory Standards Institute [6]. Time kill studies to determine the bactericidal activity of daptomycin 1 μg/ml or vancomycin 2 μg/ml alone and in combination with dexamethasone 100 μg/ml were performed as described by Anhalt and Washington [8].

### Pharmacokinetics of vancomycin and daptomycin

The 30 minute serum concentration of vancomycin or daptomycin in 25–30 gram immunocompetent hairless mice (Charles River Laboratories, Wilmington, MA) was determined following intraperitoneal injection of 40, 50 and 60 mg/kg vancomycin or 20, 30, 40 and 50 mg/kg daptomycin, respectively. Pharmacokinetic profiles were determined by injecting 50 mg/kg vancomycin or 25 mg/kg daptomycin intraperitoneally and obtaining serum samples 30, 60, 120, 180 and 240 minutes post injection. Drug activity was measured using a microbiological assay technique. Standard curves for serum drug level determination were performed by making drug dilutions in pooled mouse serum (Innovative Research, Incorporated, Southfield, MI). The reporter bacteria for the vancomycin and daptomycin bioassays were *Bacillus subtilis *and *Micrococcus luteus*, respectively. Zones of growth inhibition were measured to the nearest millimeter. The detection limit for both drugs was 0.5 μg/ml.

### Murine meningitis model

This study was approved by the Institutional Animal Care and Use Committee of the Mayo Clinic, Rochester, Minnesota. Ninety-six 25–30 gram immunocompetent hairless mice were divided into three treatment groups. Sixteen hours prior to treatment, mice were anesthetized using ketamine and xylazine (45 mg/kg and 5 mg/kg I.M., respectively) and 3 × 10^4 ^cfu of bacteria in a 20 μl volume were inoculated into the cisterna magna. The time of treatment was selected as the latest time point at which antimicrobial treatment could realistically lead to recovery. Treatment was initiated by dividing all inoculated mice into three groups and treating with 1 mg/kg dexamethasone alone, or in combination with 25 mg/kg daptomycin or 50 mg/kg vancomycin. At 22 hours post infection an additional dose of dexamethasone 1 mg/kg was administered. At 20 hours post infection an additional dose of vancomycin 50 mg/kg was given to the vancomycin-treated animals. Mice not receiving vancomycin at 20 hours were injected with an equal volume of saline. At 16, 20, and 24 hours post infection, 8, 40 and 48 animals, respectively were re anesthetized and cerebrospinal fluid (CSF) was collected by way of puncture from the cisterna magna (using a 28 G 1/2 needle 0.3 × 13 mm from BD Microlance). CSF white-blood cell count was determined and bacterial burden was quantified in CSF by plating serial 10-fold dilutions in sterile isotonic saline onto blood agar plates. Results were expressed as log_10 _cfu/ml; the detection limit was 20 cfu/ml of CSF. Following collection of CSF, animals were euthanized with a lethal dose of pentobarbital, their brains collected and placed in 10% neutral buffered formalin. There was no mortality (prior to tissue harvest).

### Imaging studies

In vivo bioluminescence imaging was performed using the Lumazone Imaging System (1002 FE series; Roper Scientific, Tucson, Arizona) at 16, 20 and 24 hours post infection. Animals were sedated with ketamine plus xylazine, placed in an imaging box without restraint, and imaged for a maximum of 10 minutes at 4 × 4 binning resolution. Luminescence was quantified in photons/sec; correlation analysis with bacterial titers was performed.

### Histopathology studies

Serial coronal sections of formalin-fixed whole brain were prepared. Brain was routinely processed, embedded in paraffin, sectioned at approximately 5–6 μm, and stained with hematoxylin and eosin (H&E), cresyl echt violet for Nissl substance, and a rabbit anti-human cleaved caspase-3 HRP antibody (Seventh Wave Laboratories, Chesterfield, MO), for apoptosis. The whole brain sections were scored for inflammation, neuronal necrosis, and parenchymal apoptosis by an independent veterinary pathologist (Dr. Ewing, Genzyme Corporation, Cambridge, MA).

### Statistics

Bacterial concentrations between treatment groups at each time point were compared using the student t-test. Correlation analysis between log_10 _bacterial CSF titers and log_10 _photons/second was calculated using SPSS statistical software. For inflammation, neuronal necrosis, and apoptosis scores, statistical analysis included a nonparametric Kruskal-Wallis test followed by a Dunn's test comparing all treatment groups using a confidence level of 95%.

## Results

### In vitro studies

**Figure 1:**
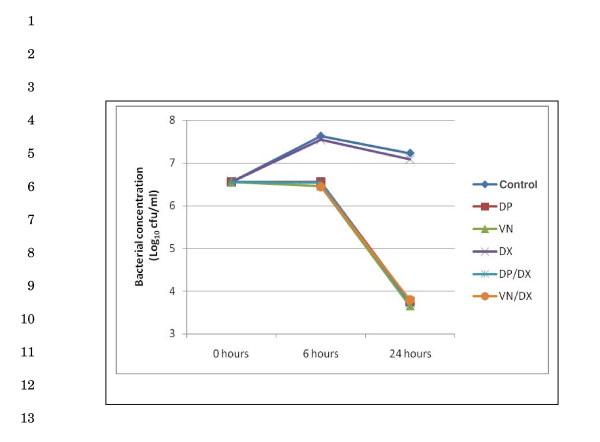
**Time kill assay**. Bactericidal activities of daptomycin (1 μg/ml) or vancomycin (2 μg/ml) are not affected by dexamethasone (100 μg/ml). DP, daptomycin; VN, vancomycin; DX, dexamethasone.

The MIC/MBC of daptomycin, vancomycin, penicillin and dexamethasone were 0.06/0.125, 0.125/0.25, 0.03/0.06 and > 128/> 128 respectively. The *in vitro *bactericidal activity of daptomycin and vancomycin was not affected by 100 μg/ml of dexamethasone (Figure 1).

### Pharmacokinetics of vancomycin and daptomycin

**Figure 2:**
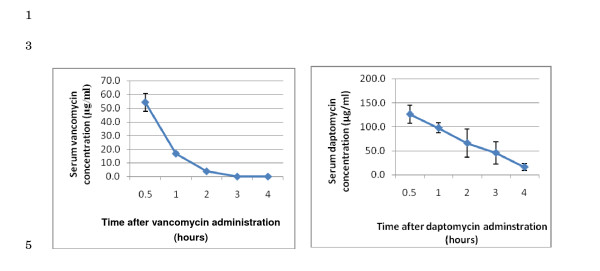
**Serum antibiotic concentration after a single intraperitoneal dose of vancomycin (50 mg/kg) or daptomycin (25 mg/kg)**.

The highest dose of daptomycin currently approved by the FDA is 6 mg/kg q.d. intravenously, corresponding to a serum C_max _of 90–100 μg/ml. In mice, 25 mg/kg of daptomycin intraperitoneally was found to best approximate the human C_max _and used for the remainder of the experiments (Figure 2). A 30 minute daptomycin concentration of 127 μg/ml decreased to 16 μg/ml after 4 hours. Vancomycin was given at a dose of 50 mg/kg which yielded a level of 55 μg/ml at 30 minutes, thereafter rapidly declining to < 0.5 μg/ml at 3 hours (Figure 2). Because of its short half-life, vancomycin was given every 4 hours, whereas daptomycin was given once. Dexamethasone was given every 6 hours.

### Murine meningitis model

**Figure 3:**
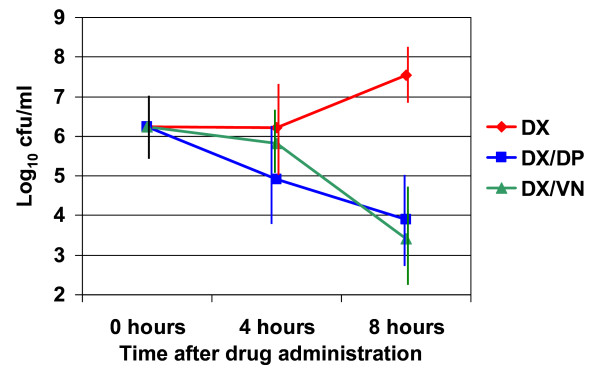
**Concentrations of *Streptococcus pneumoniae *in cerebrospinal fluid**. Concentration of *S. pneumoniae *in cerebrospinal fluid after 0, 4 and 8 hours of intraperitoneal injection of dexamethasone (administered at 0 and 6 hours) alone (DX), daptomycin (administered at 0 hours) and dexamethasone (administered at 0 and 6 hours) (DX/DP), or vancomycin (administered at 0 and 4 hours) and dexamethasone (administered at 0 and 6 hours) (DX/VN).

Daptomycin and vancomycin, given in combination with dexamethasone, resulted in similar bactericidal activity in vivo. Mice treated with dexamethasone alone showed an increase in CSF pneumococcal concentration after 8 hours (+ 1.31 log_10 _cfu/ml), but not after 4 hours (- 0.02 log_10 _cfu/ml) of treatment (Table 1, Figure 3). Four hours after a single treatment with either daptomycin or vancomycin accompanied by 1 mg/kg of dexamethasone, bacterial titers dropped by 1.33 and 0.43 log_10 _respectively (p-value: 0.004 and 0.36, respectively, versus no antibiotic). After 8 hours, daptomycin-/dexamethasone- and vancomycin-/dexamethasone-treated mice showed a reduction of bacterial titer of 2.33 log_10 _and 2.82 log_10 _versus no antibiotic (p-value < 0.001 for both groups). The antibiotic treatment groups differed significantly at 4 (p-value 0.02 daptomycin versus vancomycin), but not at 8 (p-value 0.18, daptomycin versus vancomycin) hours. At 8 hours, 2/16 of the daptomycin-/dexamethasone-treated mice and 4/16 of the vancomycin-/dexamethasone-treated mice had bacterial concentrations below the limit of detection.

**Table 1:** Mean bacterial concentration at time of treatment (0 hours ± SD, pooled data from all three groups), and after 4 or 8 hours of treatment respectively, expressed in log_10 _cfu/ml.

Treatment	0 hours (log_10 _cfu/ml)	4 hours (Δlog_10 _cfu/ml)	8 hours (Δlog_10 _cfu/ml)
Dexamethasone		-0.02 ± 1.49	1.31 ± 1.16
Dexamethasone/daptomycin*	6.24 ± 0.86	-1.33 ± 1.24	-2.33 ± 1.51
Dexamethasone/vancomycin^#^		-0.43 ± 1.49	-2.82 ± 1.36

*Daptomycin 25 mg/kg intraperitoneally, administered at 0 hours.

^#^Vancomycin 50 mg/kg intraperitoneally, administered at 0 and 4 hours.

White blood cell quantification in the CSF appeared to be influenced by blood contamination during sample collection. No significant difference between treatment groups was observed at any time point (data not shown).

### Imaging studies

**Figure 4:**
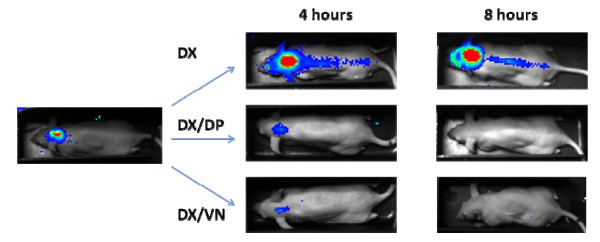
**Representative bioluminescence images**. Representative bioluminescence images taken using the Lumazone Imaging System after 0, 4 and 8 hours of treatment with dexamethasone (administered at 0 and 6 hours) alone (DX), daptomycin (administered at 0 hours) and dexamethasone (administered at 0 and 6 hours) (DX/DP), or vancomycin (administered at 0 and 4 hours) and dexamethasone (administered at 0 and 6 hours) (DX/VN). Photons/second are displayed on a calibrated color overlay (blue = low, through red = high).

**Figure 5:**
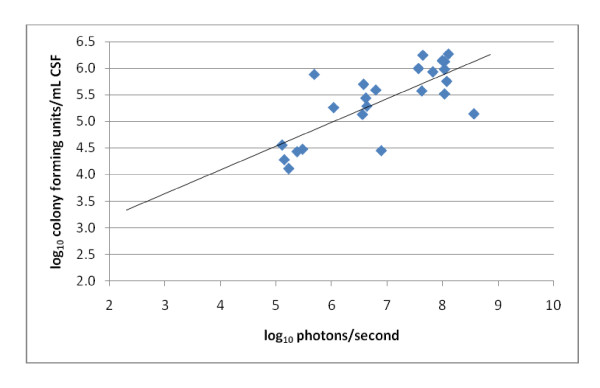
**Correlation between bacterial cerebrospinal fluid concentration and chemiluminescence (Pearson's R, 0.75; P < 0.0001)**.

Due to technical problems with the imaging system, 70 of 96 (73%) mice were imaged. Forty-six mice had luminescence lower than the detection limit and were not included in the correlation analysis. Amongst the 24 animals with luminescence above the detection limit, imaging findings were consistent with meningitis without infection of other sites (Figure 4). Imaging studies revealed a correlation (Pearson's R) of 0.75 between CSF bacterial titer and photons per second (P < 0.0001). The luminescence detection limit corresponded to 4.0 log_10 _cfu/ml or 5.0 log_10 _photons/sec, when imaging for 10 minutes at 4 × 4 binning resolution (Figure 5).

### Histopathology studies

**Figure 6:**
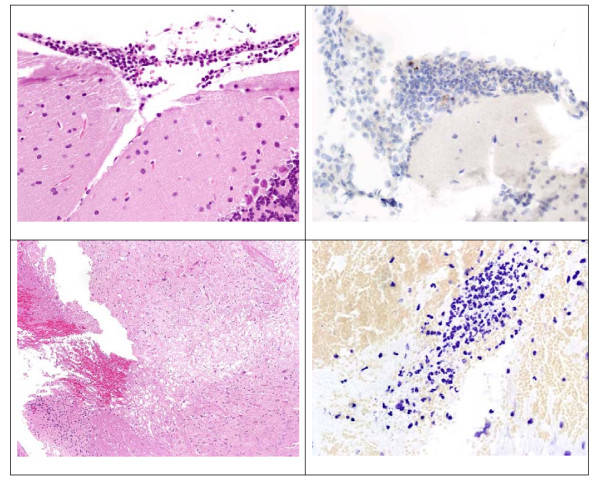
**Photomicrographs of brain from pre-treatment (16 hours) mice**. Upper panels; H&E 400× left, caspase-3 400× right, and lower panels; H&E 400× left; cresyl echt violet 500× right. Meninges of the cerebellum showed a mild infiltrate of neutrophils (upper left panel), exhibiting multifocal positivity with the apoptosis marker (upper right panel). Foci of necrosis with hemorrhage in the brain stem (lower left panel), focally infiltrated by neutrophils and associated with bacterial cocci (lower right panel), were noted.

Mice intracisternally inoculated with *S. pneumoniae *had minimal to mild, focal to multifocal meningeal infiltrates of predominantly neutrophils admixed with small numbers of macrophages and small lymphocytes at one or more levels of the brain at all time points examined (Figure 6). Inflammatory cell infiltrates displayed mild multifocal apoptosis. The amounts of inflammation and apoptosis of brain parenchymal cells were equivalent among treatment groups (data not shown).

Some of the mice had involvement of the brain parenchyma primarily limited to the brainstem. The involvement featured focal neutrophilic encephalitis or abscess formation associated with variable numbers of bacterial cocci, neuronal necrosis or loss, hemorrhage, and vacuolation of the neuropil. The density of bacterial cocci in some of the foci of encephalitis of antibiotic-treated mice appeared to be lower than that of the pre-treatment or dexamethasone only treated mice. Neuronal necrosis was not observed in any other areas of the brain. The incidence of encephalitis in the dexamethasone alone, daptomycin and vancomycin groups at 20 hours was 4/8 (50%), 4/14 (29%), and 3/15 (20%), respectively (P > 0.05). The incidence of encephalitis in the dexamethasone alone, daptomycin and vancomycin groups at 24 hours was 5/10 (50%), 2/15 (13%), and 5/14 (36%), respectively (P > 0.05).

## Discussion

At drug serum concentrations approximating the human C_max_, daptomycin and vancomycin were both highly effective at killing *S. pneumoniae *in CSF, when given in combination with dexamethasone. Daptomycin was more rapidly bactericidal than vancomycin, resulting in a significantly lower bacterial CSF titer after just four hours of treatment, although after eight hours, CSF bacterial counts did not differ significantly. *In vitro *time-kill studies demonstrated equivalent bactericidal activity of daptomycin and vancomycin.

Four studies of daptomycin treatment of experimental meningitis have been reported [8-11]. Using a rabbit meningitis model, Cottagnoud et al. showed a 6% (of serum drug levels) penetration of daptomycin across inflamed meninges, and better activity than ceftriaxone plus vancomycin against penicillin-resistant pneumococcal strains [8]. A follow-up study showed that daptomycin was associated with negligible release of [^3^H]choline (a marker of cell wall lysis) compared with ceftriaxone treatment [10]. Similarly, Gerber et al. reported bactericidal activity in a rabbit model of *Staphylococcus aureus *meningitis; they also observed that the degree of meningeal inflammation affected the penetration of daptomycin across the blood-brain-barrier [9]. Finally, Grandgirard et al. compared daptomycin with ceftriaxone in an experimental model of rat pneumococcal meningitis; daptomycin more efficiently cleared pneumococci from CSF than did ceftriaxone, reduced the concentration of matrix metalloproteinase-9 concentration in CSF 40 hours after infection, and prevented development of cortical injury [11]. None of these studies used dexamethasone as adjuvant treatment, which has become standard practice in the treatment of human bacterial meningitis in adults in developed countries, and which may impact the activity of antimicrobial agents in meningitis [12,13].

The pharmacokinetic and pharmacodynamic properties of both vancomycin and daptomycin have been described in previous studies, and have been shown to vary considerably between animal models. Based on the pharmacokinetic characteristics in our mouse meningitis model, our best approximation of human pharmacokinetic parameters was achieved by giving daptomycin once and vancomycin twice, four hours apart. Vancomycin was rapidly cleared from the serum of the mice in our study. Thus, at the four hour time point, all treatment groups had received a single dose of antimicrobial. At the 8 hour time point, the vancomycin treated group had received two doses of vancomycin, and all mice had received a second dose of dexamethasone. Although pharmacokinetic approximation is clearly a limitation of animal studies, careful conclusions about the efficacy of both drugs in our model can still be made.

The use of bioluminescent *S. pneumoniae *and the Lumazone imaging system allow for real-time, non-invasive determination of bacterial activity in CSF as a measure of infection and treatment thereof, thus limiting the need for multiple invasive CSF withdrawals per animal, and reducing the number of required animals per treatment group. However, the bioluminescent detection limit corresponded to 4 log_10 _cfu/ml, almost 2 log_10 _higher than the detection limit using quantitative cultures (2.3 log_10 _cfu/ml), making luminescent imaging most useful during the initial phase of the infection. Although bioluminescent data was not obtained on all animals in this study, animals with the complete spectrum of bacterial CSF concentrations were imaged. Because bioluminescence measured the activity of bacteria rather than actual bacterial killing, no definite conclusions can be inferred regarding the bactericidal versus bacteriostatic effects of the antimicrobial treatment regimens. Nevertheless, *in vivo *photonic imaging remains a relevant method for studying the pathogenesis and pathophysiology of bacterial meningitis.

Clinical trials of daptomycin for the treatment of Gram-positive bacteremia, endocarditis, and complicated skin and skin-structure infections have yielded favorable results [2,3,14]. A case report of successful treatment of methicillin-resistant *S. aureus *meningitis with daptomycin has been reported [15]. *In vitro *and animal model studies have shown diminished inflammatory response to infections treated with daptomycin compared with comparators [13,16]. The lack of noted differences in inflammation among treatment groups that had statistically significantly lower mean bacterial titers (i.e., daptomycin and vancomycin groups compared to no antibiotic groups) may be related to the relatively early time point of collection of brains, and/or to the concomitant administration of dexamethasone. Histopathologic evaluation of brains at later time points may be required to appreciate treatment group differences in inflammation. Further animal studies are necessary to investigate the effects of daptomycin/dexamethasone treatment on outcome parameters such as CSF inflammation, brain tissue damage and residual neurological deficit, in the context of dexamethasone treatment.

## Conclusion

Daptomycin or vancomycin, when given in combination with dexamethasone, is active in the treatment of experimental pneumococcal meningitis. The observed bactericidal activity in this study is consistent with previous studies and provides support for future evaluation of daptomycin as an alternative to vancomycin in the treatment of bacterial meningitis.

## Competing interests

The authors declare that they have no competing interests.

## Authors' contributions

BBM carried out the animal studies, compiled, analyzed and interpreted the data, and drafted the manuscript. MSR carried out the animal studies, and participated in data analysis and interpretation. CK participated in the animal studies. DVB conceived of and designed the study, and participated in data analysis and interpretation. JMS participated in data analysis and interpretation. RP participated in the design and coordination of the study, analysis and interpretation of data, and manuscript preparation. All authors read and approved the final manuscript.

## Pre-publication history

The pre-publication history for this paper can be accessed here:

http://www.biomedcentral.com/1471-2334/9/50/prepub

## References

[B1] Beek D van de de Gans J Tunkel AR Community-acquired bacterial meningitis in adults N Engl J Med 2006 354 44 53 10.1056/NEJMra052116 16394301

[B2] Carpenter CF Chambers HF Daptomycin: another novel agent for treating infections due to drug-resistant gram-positive pathogens Clin Infect Dis 2004 38 994 1000 10.1086/383472 15034832

[B3] Rybak MJ The efficacy and safety of daptomycin: first in a new class of antibiotics for Gram-positive bacteria Clin Microbiol Infect 2006 12 Suppl 1 24 32 10.1111/j.1469-0691.2006.01342.x 16445721

[B4] Francis KP Yu J Bellinger-Kawahara C Visualizing pneumococcal infections in the lungs of live mice using bioluminescent *Streptococcus pneumoniae *transformed with a novel gram-positive *lux *transposon Infect Immun 2001 69 3350 8 11292758 10.1128/IAI.69.5.3350-3358.2001 PMC98294

[B5] Beek D van de de Gans J Spanjaard L Clinical features and prognostic factors in adults with bacterial meningitis N Engl J Med 2004 351 1849 59 10.1056/NEJMoa040845 15509818

[B6] Clinical and Laboratory Standards Institute Methods for dilution antimicrobial susceptibility tests for bacteria that grow aerobically; approved standard Wayne 2006 M07-A7

[B7] Anhalt JP Washington JA Bacterial Tests, Laboratory Procedures in Clinical Microbiology 1985 2 Springer Verlag, New York, NY 2731 745

[B8] Cottagnoud P Pfister M Acosta F Daptomycin is highly efficacious against penicillin-resistant and penicillin- and quinolone-resistant pneumococci in experimental meningitis Antimicrob Agents Chemother 2004 48 3928 33 15388454 10.1128/AAC.48.10.3928-3933.2004 PMC521930

[B9] Gerber P Stucki A Acosta F Daptomycin is more efficacious than vancomycin against a methicillin-susceptible *Staphylococcus aureus *in experimental meningitis J Antimicrob Chemother 2006 57 720 3 10.1093/jac/dkl007 16459345

[B10] Stucki A Cottagnoud M Winkelmann V Daptomycin produces an enhanced bactericidal activity compared to ceftriaxone, measured by [3H]choline release in the cerebrospinal fluid, in experimental meningitis due to a penicillin-resistant pneumococcal strain without lysing its cell wall Antimicrob Agents Chemother 2007 51 2249 52 17371817 10.1128/AAC.01000-06 PMC1891375

[B11] Grandgirard D Schurch C Cottagnoud P Prevention of brain injury by the nonbacteriolytic antibiotic daptomycin in experimental pneumococcal meningitis Antimicrob Agents Chemother 2007 51 2173 8 17371820 10.1128/AAC.01014-06 PMC1891377

[B12] Paris MM Hickey SM Uscher MI Effect of dexamethasone on therapy of experimental penicillin- and cephalosporin-resistant pneumococcal meningitis Antimicrob Agents Chemother 1994 38 1320 4 8092832 10.1128/aac.38.6.1320 PMC188205

[B13] Cabellos C Martinez-Lacasa J Martos A Influence of dexamethasone on efficacy of ceftriaxone and vancomycin therapy in experimental pneumococcal meningitis Antimicrob Agents Chemother 1995 39 2158 60 8540738 10.1128/aac.39.9.2158 PMC162903

[B14] Arbeit RD Maki D Tally FP The safety and efficacy of daptomycin for the treatment of complicated skin and skin-structure infections Clin Infect Dis 2004 38 1673 81 10.1086/420818 15227611

[B15] Lee DH Palermo B Chowdhury M Successful treatment of methicillin-resistant *Staphylococcus aureus *meningitis with daptomycin Clin Infect Dis 2008 47 588 90 10.1086/590257 18636964

[B16] English BK Maryniw EM Talati AJ Diminished macrophage inflammatory response to *Staphylococcus aureus *isolates exposed to daptomycin versus vancomycin or oxacillin Antimicrob Agents Chemother 2006 50 2225 7 16723590 10.1128/AAC.01559-05 PMC1479096

